# The first complete chloroplast genome of *Hovenia acerba* Lindl.

**DOI:** 10.1080/23802359.2019.1711234

**Published:** 2020-01-16

**Authors:** Yanlei Yin, Jihan Tao, Xuemei Yang, Qiqing Jiao, Yingpeng Li, Lijuan Feng

**Affiliations:** a Shandong Institute of Pomology, Taian, China;; b Dianzi Forestry Station of Guanxian County, Liaocheng, Shandong, China

**Keywords:** *Hovenia acerba*, Illumina sequencing, Chloroplast genome, Phylogenetic relationship

## Abstract

*Hovenia acerba* Lindl. is an important medicinal plant, for which complete chloroplast genome (Accession: MN782301) was sequenced, assembled and annotated. The genome size is 161,668 bp and the overall GC content is 36.69%, with large single-copy (LSC, 89,451bp) regions, small single-copy (SSC, 18,979 bp) regions, and two inverted repeat regions (IRs, 26,619 bp each). A total of 130 genes are successfully annotated, including 85 protein-coding genes, 37 tRNA genes, and 8 rRNA genes. The phylogenetic relationships showed that *H. acerba* is closely related to the species of *Ziziphus* genus.

*Hovenia acerba* Lindl. is a medicinal plant, belonging to *Hovenia* of Rhamnaceae family (Xu et al. 2012). Its pedicle is fleshy and fat, sweet in taste, and edible in a mature period. It is widely cultivated in many regions of China, such as Shanxi, Sichuan, Chongqing, Zhejiang, Shandong, etc. (Liu et al. 2017). It has antioxidant, antineoplastic, and hangover properties, and has been used broadly in medical research (Xiang et al. 2011; Zhang et al. 2019). The complete chloroplast (cp) genome can provide valuable genomic information for the conservation and phylogenetic studies of valuable species (Liu et al. 2019). However, the cp genome of *H. acerba* Lindl. has not been fully sequenced. In this present study, we reported and characterized the complete chloroplast (cp) genome of *H. acerba* based on Illumina pair-end sequencing and compared it with other genus chloroplast genome sequences. It is helpful for the evolution process and genetics conservation of *Hovenia* genus.

The fresh leaves of *H. acerba* Lindl. (Voucher specimen Accession No. SDGZ0018) were collected from the Wanjishan field of Shandong Institute of Pomology (36.21°N, 117.08°E), Shandong Province, China. Total genomic DNA was extracted using the DNeasy Plant Mini Kit (Qiagen, Venlo, Netherlands). cpDNA sequencing was performed with an Illumina Hiseq 2500 platform by Nanjing Genepioneer Biotechnologies. The fastp program was used to filter the raw paired-end reads of cpDNA (Chen et al. 2018). The GetOrganelle was used to perform *de novo* assembly (Jin et al. 2018). The cp genome was annotated using the program DOGMA (Wyman et al. 2004). The OGDRAW was used to generate the circular genome map of the genome (Lohse et al. 2013). The complete cp genome of *H. acerba* was deposited in the GenBank (Accession: MN782301).

The complete cp genome of *H. acerba* was 161,668 bp in length with an overall GC content of 36.69%. The cp genome is made up of a large single-copy region (LSC) of 89,451 bp, small single-copy region (SSC) with 18,979bp, and two inverted repeat regions (IRs) with 26,619 bp. The whole complete cp genome encoded 130 unique genes, which contained 85 protein-coding genes, 37 transfer RNA (tRNA) genes, and 8 ribosomal RNA (rRNA) genes. The tRNA genes are distributed throughout the whole genome with 22 in the LSC, one in the SSC, and 14 in the IR regions, while rRNAs are only situated in the IR regions. There were 18 duplicate genes in IRs, including seven protein-coding genes (rpl2, rpl23, ycf2, ycf15, ndhB, rps7, rps12), seven tRNA genes (trnICAU, trnL-CAA, trnV-GAC, trnI-GAU, trnA-UGC, trnRACG, trnN-GUU), and four rRNA genes (rrn16S, rrn23S, rrn4.5S, rrn5S). Among the protein-coding genes, three genes (clpP, rps12, ycf3) contained two introns, and other nine genes (atpF, ndhA, ndhB, petB, petD, rp116, rpl2, rpoC1, rps16) had one intron each.

To ascertain the phylogenetic position of *H. acerba*, 18 complete cp genomes within Rhamnaceae family were selected, *Juglans regia* (GenBank Accession No. MF167463.1) and *Diospyros maclurei* (GenBank Accession No. MH778101.1) were considered as outgroup. The cp genomes of these species were aligned using MAFFT v7.3 (Kazutaka and Standley 2013). The maximum likelihood (ML) phylogenetic tree was constructed by the IQ-TREE with the best-fit model identified using ModelFinder (Kalyaanamoorthy et al. 2017). The result showed that *H. acerba* is closely related to the species of Ziziphus genus (Figure 1). It will be valuable for the genetic study on the *Hovenia* and *Ziziphus* genus in Rhamnaceae.

**Figure 1.**
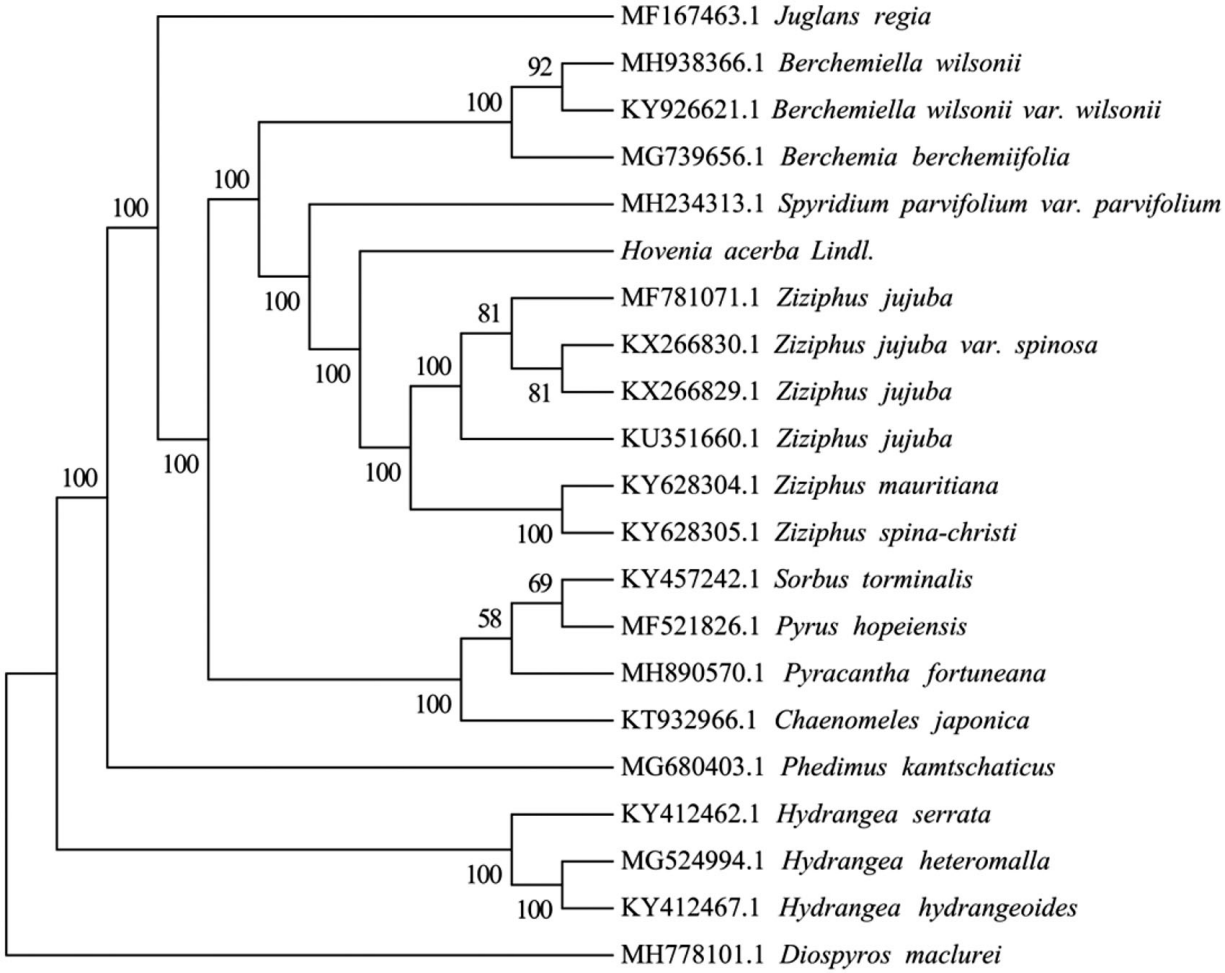
The best maximum likelihood (ML) phylogenetic tree based on the 20 complete chloroplast genome sequences. The number on each node indicates bootstrap support value.
